# A Mathematical Model Based on Stratifying the Severity of Medical Errors for Building Scenarios for Clinical Cases With Branching

**DOI:** 10.7759/cureus.58089

**Published:** 2024-04-11

**Authors:** Nataliia Lopina

**Affiliations:** 1 Simulation Training Platform "ClinCaseQuest", Med Inform Group LLC, Kharkiv, UKR

**Keywords:** clinical simulation, competency-based learning, evaluation, assessment, score system, medication errors, medical errors, branching clinical cases, branching simulation, mathematical model

## Abstract

Background

There are no mathematical models or score systems available for assessing and creating clinical case simulations based on branching scenario scripts.

Objective

This study aimed to develop a mathematical model based on stratifying the severity of medical errors for building clinical cases with branching scenarios for clinical simulation.

Methods

This study was undertaken from August 2020 to August 2023. To build a mathematical model for building scenarios of clinical cases with branching, the classification of the seriousness of medication errors was used. A mathematical model was built for predicting and modeling the development of a clinical situation and as an assessment strategy. The study recruited a total of 34 participants, with 16 participants assigned to the branching scenarios without the mathematical model group and 18 participants assigned to the branching scenarios with the mathematical model group.

Results

A simple diagram of score based on stratification of the severity of medical errors and correct decisions in clinical practice for building interactive training scenarios with branching was proposed. According to this score system algorithm, each clinical decision-making step is scored points with plus or minus, from 0 to 10. The sum of the points for each block in the decision-making process is then added up. Each step in the overall clinical decision-making strategy is stratified by the proposed algorithm, and finally, the results of internal validation and implementation are presented.

Conclusion

A mathematical model and score system for building clinical case scenarios based on branching and classification of the seriousness of medication errors was developed. This system could help in the prediction and modeling of the development of events in particular clinical situations and the assessment of competency formation in medical simulation as well.

## Introduction

Branching simulations are interactive tools that allow medical students to make decisions and observe the outcomes in simulated clinical scenarios. These simulations mimic real-life patient encounters and can be tailored to different competency levels. As students progress, the scenarios become more complex, reflecting the challenges they will face in clinical practice [1].

By engaging in branching simulations, students can develop critical thinking skills and learn to make informed decisions under pressure. These experiences bridge the gap between theoretical knowledge and practical application, better preparing future healthcare professionals to handle clinical cases effectively [2].

Branching clinical case simulations offer a dynamic and adaptable approach to skill development, fostering competencies such as critical thinking, decision-making, and clinical reasoning [3].

A number of mathematical models and methodologies are commonly used for clinical simulation assessment [4-14], but there are no mathematical models or score systems available for assessing and creating clinical case simulations based on branching, according to a PubMed and Cochrane Library search.

There are thus no available mathematical models or score systems for assessment and creating clinical case simulations based on branching scenario scripts.

To create simulation scenarios based on branching, one of the best strategies is stratifying medical errors as a model for predicting and modeling the development of clinical situations, depending on the choice of the student, and as an assessment tool.

This article presents an approach for building a mathematical model - a specific score system - based on stratifying medical errors for building clinical cases with branching scenarios.

## Materials and methods

This study was conducted from August 2020 to August 2023. The research aimed to develop a mathematical model based on stratifying medical errors for building scenarios of clinical cases with branching for clinical simulation and to combine the feedback of trainees from the branching simulation based on the mathematical model with branching simulation and without a mathematical model.

The study was conducted at the Simulation Training Platform for continuous medical education, "ClinCaseQuest", аs an internal validation experiment.

Healthcare professionals were included as medical trainees of the platform. The inclusion criteria for participation in the study comprised healthcare professionals who voluntarily consented to take part after registration in the Ukrainian version of the platform. Among 2089 registered users of the platform from different hospitals and institutions, information consent was received from 34 users. There were no additional criteria for inclusion or exclusion.

A total of 34 participants - healthcare professionals - were recruited for this study. The participants were randomly assigned to two groups: one experiencing branching clinical case scenarios without the mathematical model (n = 16) and the other using the mathematical model (n = 18). All participants were healthcare professionals from different educational institutions and hospitals in Ukraine. Also included were two instructors, each one for a separate group. The platform includes a total of 10 medical educators. For this study, two of them agreed to participate as instructors. The participants in the two groups were comparable in gender.

The study was conducted following ethical principles following the Declaration of Helsinki. Informed consent was obtained from all participants. 

Appendix A (see Appendices) presents all the steps of the implementation and feasibility of the mathematical model in the simulation training.

Appendix B (see Appendices) contains a list of clinical case scenarios used in the internal pedagogical research experiment. The pedagogical experiment employed a mixed-methods approach, incorporating both quantitative and qualitative measures to assess the effectiveness and feasibility of the mathematical model in the simulation training. This study was designed to evaluate the impact of integrating a mathematical model based on stratifying medical errors into the development of branching clinical case scenarios.

A literature search based on PubMed, Cochrane Library, ERIC, and PsycINFO was provided, employing the search terms and concepts and their Boolean combinations: a mathematical model for building scenarios of clinical cases with branching and a score system for branching simulation. Hand searching and internet searches were also used. After an exhaustive search, no available mathematical models or score systems for assessing and creating clinical case simulations based on branching scenario scripts were found.

Medical errors were chosen as a potential base for building a mathematical model for building scenarios of clinical cases with branching and as a prediction of events in each clinical case scenario, depending on the learner’s choice.

One of the most appropriate classifications for creating a branching simulation is the National Coordinating Council for Medication Error Reporting and Prevention's (NCCMERP) classification of the seriousness of medication errors. This classification not only serves as a foundation for medication errors but can also be effectively applied to assess medical errors in general. Evaluating medical errors against the same parameters as medication errors, including error occurrence, reaching the patient, associated harm, and necessary measures, then becomes paramount. The NCCMERP classification stands out as the best and most accurate choice for this purpose (Table 1) [15].

**Table 1:** Classification of the seriousness of medication errors according to the NCCMERP. Harm: Impairment of the physical, emotional, or psychological function or structure of the body and/or pain resulting therefrom. Monitoring: To observe or record relevant physiological or psychological signs. Intervention: This may include changes in therapy or active medical/surgical treatment. Vital support intervention (intervention necessary to sustain life): This includes cardiovascular and respiratory support (e.g., CPR, defibrillation, intubation). Permission granted by the National Coordinating Council for Medication Error Reporting and Prevention (NCCMERP) [15].

Error Category	Error Occurrence	Reached Patient	Associated Harm	Necessary Measures
A	Potential	No	No	No
B	Yes	No	No	No
C	Yes	Yes	No	No
D	Yes	Yes	No	Monitoring
E	Yes	Yes	Temporary	Medical intervention
F	Yes	Yes	Temporary	Hospitalization or prolonging hospital stay
G	Yes	Yes	Permanent	Variable
H	Yes	Yes	Risk of death	Vital support intervention
I	Yes	Yes	Death	-

Seriousness is categorized using nine different categories, from A to I. The classification includes the occurrence of an error, whether the error reached the patient or not, the harm associated with the error, and the necessary measures. The classification was based on the National Coordinating Council for Medication.

This system could be utilized to construct clinical case scenarios with branching as a scoring system for clinical prediction and modeling of the development of a clinical situation not only to stratify medication errors but also to categorize medical errors in general at each stage of clinical decision-making, as well as assess competency formation in simulation training.

Implementation and feasibility of the mathematical model in simulation training for continuous medical education (undergraduate and postgraduate) were performed via six steps: scenario script writing, branching scenario development without the mathematical model using program tools, mathematical model development based on the stratification of the medical errors for building branching simulation, and branching scenario development based on the mathematical model using program tools, participant access, and instructor training.

In the study evaluating the integration of a mathematical model into simulation training scenarios, participant feedback played a central role. Participants were actively engaged in providing assessments, and their responses were used to gauge the effectiveness of the simulation training.

The implementation phase involved the development of clinical case scenarios with branching. For the group without the mathematical model, scenarios were crafted using standard program tools. In contrast, the group with the mathematical model experienced scenarios based on the mathematical model’s stratification of the severity of medical errors.

Quantitative data were collected through feedback forms, where participants rated various aspects of the simulation training experience on a scale from 1 to 5. The realism of clinical scenarios, clarity of the decision-making process, integration of clinical knowledge, adaptability of scenarios, learner engagement, depth of learning from mistakes, user-friendliness of the platform, and overall satisfaction were assessed. Each component is assessed using a 5-point Likert scale, with mean values and standard deviations provided for each group. Higher mean values indicate higher levels of effectiveness or satisfaction.

Statistical analysis included the calculation of means, standard deviations, and p-values for each criterion assessed in the feedback forms. The results were compared between the group experiencing scenarios with the mathematical model and the group without, using appropriate statistical tests to determine significance.

All participants in the study completed a comprehensive feedback form, providing ratings on a scale from 1 to 5, where 5 represented the highest score for each parameter evaluated. The feedback form encompassed various aspects related to the simulation training experience, allowing participants to express their opinions and perceptions.

Quantitative data were analyzed using statistical methods, including descriptive statistics, and t-tests to compare outcomes between the experimental and control groups.

## Results

To build the mathematical model for the clinical cases with branching scenarios, the NCCMERP’s classification of the seriousness of medication errors was used [15]. A mathematical model for building scenarios of clinical cases with branching was then built to predict and model the development of clinical situations, depending on the choice of the student, as well as used as an assessment strategy.

When building a mathematical model or score system for clinical cases with branching, on the one hand, we need an integrated assessment. On the other hand, a mathematical scale that helps in building a prediction and modeling of the development of events in each specific clinical case is required.

For providing clinical prediction and modeling in branching scenarios, two additional columns could be added (points for clinical decision-making and clinical case situation development) (Table 2).

**Table 2:** Classification of the seriousness of medical errors for clinical case prediction and modeling for branching scenario script building

Error Category	Error Occurrence	Reached Patient	Associated Harm	Necessary Measures	Points for Clinical Decision-Making	Clinical Case Situation Development
A	Potential	No	No	No	0	Continue
B	Yes	No	No	No	0	Continue
C	Yes	Yes	No	No	-1	Continue
D	Yes	Yes	No	Monitoring	-2	Continue with measures for patient monitoring
E	Yes	Yes	Temporary	Medical intervention	-3	Continue with medical intervention (if the medical intervention is appropriate, the patient state improves. If not, use F-I classification – patient state getting worse and requires further steps and additional calculations)
F	Yes	Yes	Temporary	Hospitalization or prolonging hospital stay	-4	Continue with medical intervention (if the medical intervention is appropriate, the patient's state improves. If not, use F-I classification – patient state getting worse and requires further steps and additional calculations)
G	Yes	Yes	Permanent	Variable	-5	Continue with further decision-making steps with the possibility of clinical case termination (if further steps are appropriate, patient state improves. If not, use G-I classification – patient state getting worse and requires further steps and additional calculations)
H	Yes	Yes	Risk of death	Vital support intervention	-6	Continue with further decision-making steps with the possibility of clinical case termination (if further steps are appropriate, patient state improves. If not, use H-I classification – patient state getting worse and requires further steps and additional calculations)
I	Yes	Yes	Death	-	-10	Clinical scenario stop

Clinical prediction and modeling of the development of a clinical situation were also based on the literature data of the nosology presentation, available data, and similar clinical cases. Each clinical situation has a multivariate development of events, but there are always several decision-making points that accompany serious medical errors, resulting in associated harm and requiring associated measures.

**Figure 1:**
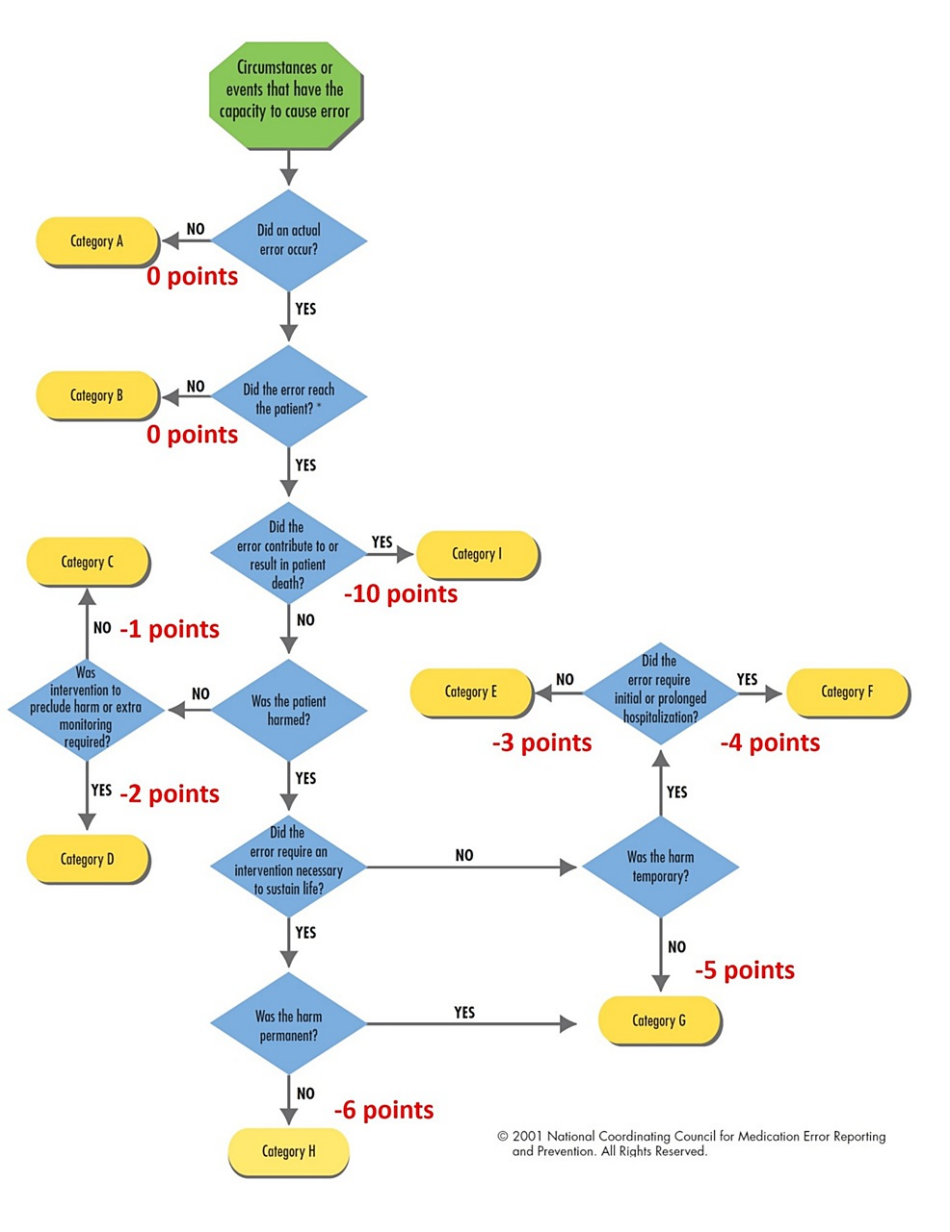
Classification of the seriousness of medication errors according to the NCCMERP, with points for building branching simulation. Permission granted by the National Coordinating Council for Medication Error Reporting and Prevention (NCCMERP).

Figure 1 provides a practical visual demonstration to enhance visibility and understanding of the classification of the seriousness of errors according to the NCCMERP, with points for building branching simulation.

For appropriate decision-making, the same classification specular to the medical errors could be used (Table 3).

**Table 3:** Point assessment for the correct decision-making process for clinical case prediction and modeling for branching scenario script building

Correct Decision-Making Category	Positive Changes Occurrence	Reached Patient	Patient State and Correct Decision-Making	Points for Clinical Decision-Making	Clinical Case Situation Development
A^/^	Potential	No	Not change	0	Continue
B^/^	Yes	No	Not change	0	Continue
C^/^	Yes	Yes	Taking the right steps toward a diagnosis and further treatment, but not directly changing patient state	1	Continue
D^/^	Yes	Yes	Making the right steps toward a diagnosis and further treatment with positive changes in the patient’s state	2	Continue
E^/^	Yes	Yes	Making the significant right steps toward a diagnosis and further treatment with positive changes in the patient’s state	3	Continue
F^/^	Yes	Yes	Correct diagnosis making	4	Continue, or successfully finish, depending on the education goals
G^/^	Yes	Yes	Choosing the right treatment strategy with improvements in the patient’s state	5	Successfully finished or proceed as a follow-up
H^/^	Yes	Yes	Choosing the right treatment strategy with significant improvement in the patient’s state	6	Successfully finished or proceed as a follow-up
I^/^	Yes	Yes	Complete recovery	10	Successfully finished or proceed as a follow-up

**Figure 2:**
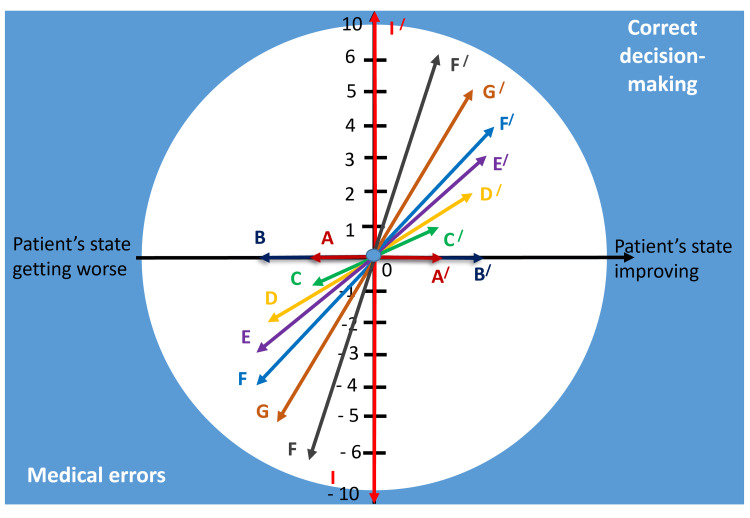
Diagram of score stratification of medical errors and correct decisions for building simulation training scenarios with branching Image credits: Nataliia Lopina

Figure 2 shows a summary diagram of the scoring stratification of the clinical decision-making process for building simulation training scenarios with branching.

The main blocks in providing medical care in the decision-making process are as follows: communication with the patient (C), objective examination of the patient (OE), differential diagnosis 1 (based on the main syndrome - DD1), lab test assignment and evaluation (LT), diagnostic tests assignment and evaluation (DT), differential diagnosis 2 (after collecting the results of the laboratory and diagnostic tests - DD2), making a diagnosis (MD), prescribing treatment (T), manual skills and procedures (MP), monitoring (M) and follow-up (F).

The sum of points for each block in the decision-making process was added up, thus obtaining the total score for completing the simulation training.

The sum of points within each block was calculated as the sum of points for the clinical decision-making strategy, with a positive or negative value (see Table 2 and Table 3). Each step in the overall clinical decision-making strategy was stratified by the proposed algorithm.

The general formula for estimating a branching scenario (total clinical decision-making score) was as follows:

$\sum = \sum C_n\dotplus \sum OE_n\dotplus \sum D1D_n \dotplus \sum LT_n \dotplus \sum DT_n\ \dotplus \sum D2D_n\ \dotplus \sum MD_n\ \dotplus \sum T_n\ \dotplus \sum MP_n\ \dotplus \sum M_n\ \dotplus \sum F_n$, where n- is the number of variables in each clinical decision block, $\sum C_n$ is the sum of points within the communication decision-making block; $\sum OE_n$ is the sum of points within the objective examination block decision-making block, $\sum D1D_n$ is the sum of points within the differential diagnosis first block (based on the main syndrome), $\sum LT_n$ is the sum of points within the laboratory tests block, $\sum DT_n$ is the sum of points within the diagnostic tests assignment and evaluation block, $\sum D2D_n$ is the sum of points within the differential diagnosis second block (based on the main syndrome, laboratory and diagnostic tests results), $\sum MD_n$ is the sum of points within the making the diagnosis block, $\sum T_n$​​​​​​​ is the sum of points within the treatment block, $\sum MP_n$​​​​​​​ is the sum of points within the manual skills and procedures block, $\sum M_n$​​​​​​​_ _is the sum of points within the monitoring block, $\sum F_n$​​​​​​​ is the sum of points within the follow-up block (if available).

Total clinical decision-making score = sum of points of the next components:

1. $\sum C_n = C_1\dotplus C_2\dotplus C_3 \dotplus C_4 \dotplus C_5\ \dotplus \ldots \dotplus C_n$, where $\sum C_n$ is the sum of points within the communication decision-making block, and $C_n$ is the components of each decision-making step in the communication decision-making block;

2. $\sum OE_n = OE_1 + OE_2 + OE_3+ OE_4 + OE_5 + \ldots + OE_n$, where $\sum OE_n$ is the sum of points within the objective examination block, and $OE_n$ is the components of each decision-making step in the objective examination block;

3. $\sum D1D_n = D1D_1 + D1D_2 + D1D_3+ D1D_4 + D1D_5 + \ldots + D1D_n$, where $\sum D1D_n$ is the sum of points within the differential diagnosis first block (based on main syndrome), $D1D_n$ is the components of each decision-making step in the differential diagnosis first block;

4. $\sum LT_n = LT_1 + LT_2 + LT_3+ LT_4 + LT_5 + \ldots + LT_n$, where $\sum LT_n$ is the sum of points within the laboratory tests block, and $LT_n$ is the components of each decision-making step in the laboratory tests block;

5. $\sum DT_n = DT_1 + DT_2 + DT_3+ DT_4 + DT_5 + \ldots + DT_n$, where $\sum DT_n$ is the sum of points within the diagnostic tests assignment and evaluation block, and $DT_n$ is the components of each decision-making step in the diagnostic tests assignment and evaluation block;

6. $\sum D2D_n = D2D_1 + D2D_2 + D2D_3+ D2D_4 + D2D_5 + \ldots + D2D_n$, where $\sum D2D_n$ is the sum of points within the differential diagnosis second block (based on all available data after laboratory and instrumental diagnostic tests), and $D2D_n$ is the components of each decision-making step in the differential diagnosis second block;

7. $\sum MD_n = MD_1 + MD_2 + MD_3+ MD_4 + MD_5 + \ldots + MD_n$, where $\sum MD_n$ is the sum of points within the making the diagnosis block, and $MD_n$ is the components of each decision-making step in the making the diagnosis block;

8.$\sum T_n = T_1 + T_2 + T_3+ T_4 + T_5 + \ldots + T_n$, where $\sum T_n$ is the sum of points within the treatment block, and $T_n$ is the components of each decision-making step in the treatment block;

9. $\sum MP_n = MP_1 + MP_2 + MP_3+ MP_4 + MP_5 + \ldots + MP_n$, where $\sum MP_n$ is the sum of points within the manual skills and procedures block, and $MP_n$ is the components of each decision-making step in the manual skills and procedures block;

10. $\sum M_n = M_1 + M_2 + M_3+ M_4 + M_5 + \ldots + M_n$, where $\sum M_n$ is the sum of points within the monitoring block, and $M_n$ is the components of each decision-making step in the monitoring block;

11. $\sum F_n = F_1 + F_2 + F_3+ F_4 + F_5 + \ldots + F_n$, where $\sum F_n$ is the sum of points within the follow-up block (if available), and $F_n$ is a component of each decision-making step in the follow-up block (if available).

This score-based assessment diagram could help with developing a predictive model of clinical situation development for building branching clinical case scenarios and can be used as an assessment tool as well.

The research findings are summarized in Table 4. Participants who experienced simulation training with the mathematical model consistently rated the realism of clinical scenarios higher compared to those without the model (4.6 ± 0.5 vs. 3.0 ± 0.73; p-value < 0.001). This suggests the participants perceived the inclusion of the mathematical model as enhancing the authenticity and life-like nature of the clinical scenarios.

**Table 4:** Comparison of simulation scenarios with and without mathematical model integration based on trainee feedback (n = 16 for branching scenarios without the mathematical model; n = 18 for branching scenarios with the mathematical model) Statistical significance was determined using a t-test to compare the means of the two groups. P-values are reported to indicate the significance of the differences observed between the groups.

Criteria	Branching Clinical Cases Scenarios without the Mathematical Model (n = 16)	Branching Clinical Cases Scenarios with the Mathematical Model (n = 18)	p-value
Realism of Clinical Scenarios	3 ± 0.73	4.6± 0.5	<0.001
Clarity of Decision-Making Process	2.5 ± 0.52	4.2 ± 0.42	<0.001
Integration of Clinical Knowledge	3.2 ± 0.4	4.5 ± 0.51	<0.001
Adaptability of Scenarios	2.2 ± 0.4	4.0 ± 0.5	<0.001
Learner Engagement	3.6 ± 0.5	4.7 ± 0.5	<0.001
Depth of Learning from Mistakes	2.5 ± 0.5	4.4 ± 0.5	<0.001
User-Friendliness of the Platform	3.0 ± 0.73	4.6 ± 0.5	<0.001
Overall Satisfaction	2.5 ± 0.52	4.7 ± 0.5	<0.001
Average Score (out of 5)	2.8	4.5	-

The clarity of the decision-making process within the simulation was notably improved for participants using the mathematical model compared to those without it (4.2 ± 0.42 vs. 2.5 ± 0.52; p-value < 0.001). This indicates the participants found the mathematical model to contribute to a clearer and more understandable decision-making process during the simulation.

Participants who underwent training with the mathematical model reported higher scores for the integration of clinical knowledge compared to those without the model (4.5 ± 0.51 vs. 3.2 ± 0.4; p-value < 0.001). This underscores the positive impact of the mathematical model on effectively incorporating clinical knowledge into the learning experience.

The adaptability of scenarios, crucial for dynamic learning experiences, was perceived more favorably by participants with the mathematical model compared to those without it (4.0 ± 0.5 vs. 2.2 ± 0.4; p-value < 0.001. This suggests participants appreciated the adaptive nature of scenarios facilitated by the mathematical model.

Participants in the group with the mathematical model reported significantly higher levels of engagement (4.7 ± 0.5) compared to the nonmodel group (3.6 ± 0.7) (p-value < 0.001). This points to the positive impact of the mathematical model on keeping participants actively involved and invested in the simulation training.

The depth of learning from mistakes was perceived to be more substantial by participants using the mathematical model compared to those without it (4.4 ± 0.5 vs. 2.5 ± 0.5; p-value < 0.001). Participants recognized the value of the mathematical model in facilitating a more profound understanding and learning from errors.

Participants in the group with the mathematical model reported higher scores regarding the user-friendliness of the simulation platform compared to the nonmodel group (4.6 ± 0.5 vs. 3.0 ± 0.73; p-value < 0.001). This indicates the mathematical model contributed to a more user-friendly and accessible simulation platform.

The overall satisfaction of participants was significantly higher in the group with the mathematical model compared to the nonmodel group (4.7 ± 0.5 vs. 2.5 ± 0.52; p-value < 0.001). This comprehensive measure reflects participants’ overall positive perceptions of the simulation training, enhanced by the mathematical model.

The median scores further emphasize the consistent positive impact of the mathematical model. Although the nonmodel group had a median score of 2.8, the model group achieved a substantially higher median score of 4.5. This reinforced the participants’ collective positive feedback regarding various aspects of the simulation training (Table 4).

Instructors assessing the simulation training with the mathematical model expressed notably more positive feedback. They highlighted the heightened realism of the clinical scenarios and the improved clarity in the decision-making process. The adaptability of scenarios, engagement of learners, and depth of learning from mistakes were consistently praised. Instructors also acknowledged the user-friendliness of the platform, noting enhanced satisfaction compared to the group without the mathematical model.

## Discussion

Clinical thinking, the ability to analyze complex clinical situations and make informed decisions, is a cornerstone of medical education. One highly effective method for fostering clinical thinking skills is learning from case scenarios based on branching [3].

Creating case scenarios based on branching for the development of clinical thinking is an effective way to engage learners and help them develop critical thinking and decision-making skills in medical or healthcare contexts. In branching case scenarios, learners make decisions at various decision points, and the scenario unfolds differently based on their choices [16].

Branching case scenarios allow learners to explore different decision pathways and consequences in the evaluation and management of chest pain. They promote critical thinking and clinical reasoning skills while offering a realistic and engaging learning experience [17,18].

Branching clinical case scenario scripts can be based on storytelling. To improve the relationship between patients and healthcare professionals, since the 1980s, American medical schools and hospitals have promoted the humanities and, more recently, so-called narrative medicine in healthcare curricula and practices [19]. According to Charon, narrative medicine is the medicine “practiced with the narrative competence to recognize, absorb, interpret, and be moved by the stories of illness” [19]. The use of narrative medicine can have positive effects on students in regard to self-reflective thinking, inpatient-healthcare provider communication, empathy, and narrative medicine writing skills [20,21,22,23].

The synergy of adaptive learning, guided simulation, multi-repetitions, and facilitated supervision within branching case scenarios elevates the development of clinical thinking in medical education. This holistic approach ensures learners receive tailored challenges, expert guidance, ample practice opportunities, and valuable feedback.

Mathematical models play a crucial role in assessing clinical simulations in medical education. These models help educators and researchers evaluate the effectiveness of simulation-based training, identify areas for improvement, and make data-driven decisions.

The mathematical models and methodologies commonly used for clinical simulation assessment in medical education [4-14] are Objective Structured Clinical Examination (OSCE) Scoring Models, Checklist Scoring Models, Global Rating Scales (GRS), Rasch Measurement Models, Simulation-Based Competency Models, Time-Series Analysis, Hierarchical Linear Models (HLM), Machine Learning and Artificial Intelligence (AI), Item Response Theory (IRT), and Bayesian Network Models.

These mathematical models and methodologies offer a wide range of tools for assessing clinical simulations in medical education. The choice of which model to use depends on the specific goals of the assessment, the nature of the simulation, and the available data.

Medical errors are a significant cause of patient harm in clinical practice. Medical errors have been widely identified as a key cause of preventable adverse events in clinical practice, with recent estimates indicating it is the third leading cause of death in the US health system [24]. This has a significant impact on the medical community, both in the United States and globally, and its recommendations helped launch a significant drive to improve quality [25]. 

Medical errors are unintended acts or omissions in health care that lead to patient harm. They can occur at various stages of the health-care process, from diagnosis and treatment to medication administration and communication. Medical errors are typically classified into several categories to better understand their causes and prevent future occurrences. Common classifications of medical errors include the following: diagnostic errors (misdiagnosis, delayed diagnosis, failure to diagnose), treatment errors (medication errors, surgical errors procedural errors, infection control errors), communication errors (miscommunication, failure to communicate), documentation errors, equipment and technical errors, transfusion errors, fall and injury errors, healthcare-associated infections, latent errors, and systemic or organizational errors [15,26,27,28,29,30]. There are several underlying issues within healthcare systems, processes, or cultures that contribute to medical errors.

The errors may be related to professional practice, healthcare products, procedures, communication problems (including prescribing, product labeling, packaging, and nomenclature), compounding, dispensing, distribution, administration, education, monitoring, and the proper use of medications [15].

A mathematical model for building scenarios of clinical cases with branching was built based on the stratification of medication errors. The results of internal implementation and validation were presented. Further evaluation of the proposed mathematical model based on the stratification of medical errors for building scenarios of clinical cases with branching is required. Further validation studies are also needed to establish the validity and reliability of the assessment tools.

Effective implementation of the mathematical model based on the stratification of medical errors for building scenarios of clinical cases with branching necessitates training for educators or facilitators. Ensuring educators are proficient in using the model and providing constructive feedback to learners is essential. However, the use of branching scenarios with assessment components raises several ethical considerations, particularly concerning learner performance evaluations. Ensuring fairness, confidentiality, and ethical conduct in the assessment processes is crucial. The long-term impact of using the proposed model on learners’ clinical skills, patient outcomes, and healthcare quality has yet to be fully explored. Longitudinal studies are needed to assess the lasting effects. Comparative studies with other educational methods for competency formation are needed. These factors should be considered in future evaluations.

The limitations of this study include conducting a test experiment on a small group of participants and applying this development within a single online medical simulation education platform. While the model offers a structured approach to scenario development, it may not completely replicate the unpredictability and variability found in real patient care settings. Additionally, the study did not evaluate the long-term retention of knowledge and skills acquired through simulation training. Future research could investigate the sustainability of learning outcomes over time and the possibilities for the integration of this mathematical model into other simulation online platforms and in onsite simulation training. 

One limitation of the study is that participants were recruited from various healthcare institutions and hospitals, potentially introducing variability that could impact the results. Another limitation of the study was the lack of collection of demographic information from participants, as the registration form did not include provisions for such data gathering.

## Conclusions

Branching scenarios offer a unique opportunity to learn from medical errors, enabling participants to repeat specific actions multiple times until competencies are thoroughly mastered. Building a mathematical model for branching clinical case scenarios, along with developing an integrated assessment and moderation system, can provide a dynamic approach to medical education, allowing learners to engage in realistic clinical decision-making while facilitating effective feedback and competency development.

Integrating a mathematical model into branching scenarios, based on the stratification of the severity of medical errors, enhances the learning experience, allowing participants to immerse themselves more fully in the learning experience, to “feel” medical errors, and extract valuable lessons from them.

## Appendices

Appendix A

**Table 5:** Implementation and feasibility of the mathematical model in simulation training for continuous medical education (undergraduate and postgraduate) “ClinCaseQuest”

Implementation Aspects	Description
1. Scenario script writing	Scenario scripts involved writing to identify the key points for the decision-making. Potential medical errors showing different severity in scenario scripts were identified.
2. Branching scenario development without the mathematical model using program tools	Clinical case scenarios with branching were developed without the mathematical model based on the stratification of the severity of medical errors.
3. Developing the mathematical model	This involved describing all the steps in the mathematical model for building branching simulation development with detailed explanations.
4. Branching scenario development based on the mathematical model using program tools	Clinical case scenarios with branching were developed using the mathematical model based on the stratification of the severity of medical errors.
5. Participant access	All participants easily accessed the simulation training platform, completing scenarios with the mathematical model and clinical scenarios without the mathematical model.
6. Instructor training	The instructors received training on the understanding of the mathematical model and total clinical decision-making score calculation algorithm of competency formation to guide participant performance.

Appendix B

**Table 6:** The list of clinical case scenarios used in the internal pedagogical research experiment OSCE: Objective Structured Clinical Examination; CNS: central nervous system; INR: international normalized ratio

Type and title of the clinical case scenario	Branching clinical cases scenarios without using Mathematical model	Branching clinical cases scenarios with using Mathematical model
1.	Clinical case simulator – A Lady with a sudden cardiac pain (unblinded title: Acute myocardial infarction in the woman with spontaneous coronary artery dissection)	Clinical case simulator – Call to Emergency Service from a patient with chest pain (OSCE station) (unblinded title: Acute myocardial infarction of the anterior wall in the man)
2	Clinical case simulator – Chronic Myeloid Leukemia and Jaundice (unblinded title: Acute Imatinib induced liver injury)	Clinical case simulator – Sudden INR increase in the women with prosthetic mitral valve taking warfarin (unblinded title: Increase in INR in a woman who drank grapefruit juice)
3	Clinical case simulator – Choice of dual antiplatelet therapy (DAPT) duration after stenting of coronary arteries in a young woman	Clinical case simulator – Patient with chest pain (on duty in the emergency department of the hospital), OSCE station (unblinded title: Increase in INR in a woman who drank grapefruit juice)
4	Clinical case simulator – Back pain in a woman (unblinded title: Benign notochord tumor)	Clinical case simulator - A patient with severe neurological symptoms in the hematology department (unblinded title: CNS injury in lymphoma patient)
5	Clinical case simulator – Pregnancy after myocardial revascularization	Clinical case simulator - Choosing a treatment strategy for a patient with abnormalities in complete blood count (unblinded title: Сhronic myeloid leukemia)
6		Clinical case simulator - From chest pain to diagnosis in a 61-year-old patient (unblinded title: Multiple myeloma)
Total amount	5	6
